# Research Support Networks for Substance Use Translational Research: A Qualitative Evaluation of the HEAL Data to Action (HD2A) Program

**DOI:** 10.21203/rs.3.rs-8340202/v1

**Published:** 2026-01-29

**Authors:** Lawrence A. Palinkas, C. Hendricks Brown, Heather J. Gotham, Emma E. McGinty, Ethan Lai, Meredith C. B. Adams, Mark P. McGovern

**Affiliations:** University of California San Diego; Northwestern University; Stanford University School of Medicine; Weill Cornell College of Medicine; University of California San Diego; Wake Forest University School of Medicine; Stanford University School of Medicine

**Keywords:** research support, health services research, pain management, opioid-related disorders, translational research, implementation

## Abstract

**Background.:**

NIH-funded research has been increasingly conducted in collaborative consortia of investigators with the support of coordinating centers. However, the extent to which such initiatives help to accomplish NIH goals and objectives with respect to the translation of research to practice remains unclear. We evaluated the progress being made by individual Helping to End Addiction Long-term^®^ Initiative or HEAL Initiative^®^ Data2Action (HD2A) Program projects targeting reduction of overdoses and improving opioid use disorder treatment and pain management and examined project leaders’ perceptions of support provided by three research support centers.

**Methods.:**

A qualitative observational study conducted as part of a mixed method formative evaluation. Data were collected from semi-structured group interviews and interview transcripts were analyzed using thematic content analysis. Principal investigators and key personnel (n = 33) for each of the 10 NIDA-funded HD2A projects annually beginning in March 2023. Members of the three support centers (n = 8) were also interviewed in January 2025.

**Results.:**

Three overall themes with several subthemes were identified: 1) accomplishments and challenges of individual projects; 2) HD2A Support Center activities; and 3) overall assessment of HD2A participation. All projects made significant progress and obtained needed support from the three research support centers. Project implementation activities were strengthened overall. Grantees that received supplemental funding for rapid data infrastructure projects benefitted from that funding. Interactions with other HD2A projects was limited but viewed favorably by all grantees. All projects were funded by a phasic NIH grant mechanism. Transitioning from the R61 planning phase to the R33 study phase was a minor challenge for some projects but successfully addressed with the assistance of NIDA personnel. Efforts to harmonize data across the projects have encountered barriers and will require additional support and guidance.

**Conclusions.:**

Initiatives to bring together diverse projects having a common aim or theme create opportunities for accessing needed support for implementation and collaboration across projects as intended. Nevertheless, greater engagement between those providing technical assistance and those receiving it and stipulating common data elements should occur prior to submission of applications for funding.

## BACKGROUND

Despite a 27% decline in 2024 compared to the previous year (1), deaths dues to overdoses of illicit and prescription opioids remain a significant public health problem in the United States (2, 3). Accelerating the implementation of evidence-based interventions is a priority for the U.S. Department of Health and Human Services’ Overdose Prevention Strategy (4). The Helping to End Addiction Long-term^®^ (HEAL) Initiative, or National Institutes of Health (NIH) HEAL Initiative^®^, is an aggressive, multi-pronged effort involving almost every NIH Institute and Center to speed scientific solutions to stem the national opioid public health crisis (5).

As part of the NIH HEAL Initiative, the National Institute on Drug Abuse (NIDA) released a set of interrelated Funding Opportunity Announcements (FOAs) in 2020 to create the HEAL Data2Action (HD2A) Program, a coordinated effort to promote the synthesis and real-world application of existing data to improve epidemiology and guide and monitor improvements in opioid use disorder treatment and pain management (6). The HD2A Program supports to kinds of projects. *Innovation Projects* are innovative approaches to using data to drive action and change in real-world settings. Efforts focus on improving data infrastructure (e.g., data capture, data timeliness, data sharing, data linkage, data utilization) and engaging stakeholders to support data-driven decision-making and real-time feedback loops that accelerate the implementation of evidence-based practices for opioid use disorder and pain management and improve overdose-related outcomes. *Acceleration Projects* develop data or methods that improve the timeliness, quality, accessibility, or usefulness of existing data ecosystems. Ultimately, these data improvements are intended to allow for faster and improved responses to address the overdose epidemic, including primary prevention, evidence-based treatment, and recovery support. Collectively, these projects were expected to improve the quality, timeliness, accessibility and usefulness of data for epidemiology and service delivery in each of the four pillars of the HHS Overdose Prevention Strategy: primary prevention, harm reduction, treatment of opioid use disorder, and recovery support (4).

The HD2A Program includes several distinct features, but four are particularly noteworthy because they represent a novel approach to conducting services research for many of the investigators awarded funding under the auspices of this program. The first distinctive feature was the use of a R61/R33 Exploratory/Developmental Phased Award mechanism (6). The R61 phase supports activities that demonstrate feasibility of bringing together relevant stakeholders and data sources for up to two years. The primary objective of the confirmatory R33 phase is to select and deploy evidence-based interventions or strategies related to service structuring or resource allocation to fill gaps in service delivery and to utilize local data to monitor improvements in overdose-related outcomes along the opioid care cascade. When transitioning to the R33 phase, recipients were required to submit a transition package that included a report describing in detail the progress towards achieving R61 milestones and a description of how research proposed for the R33 phase would be supported by the completion of these milestones. These materials were evaluated by NIH program staff to determine if the milestones were met. R33 funding decisions were then based on the original R61/R33 peer review recommendations, successful completion of transition milestones, any proposed changes to the R33 research based on R61 findings, program priorities, and availability of funds.

The second distinctive feature of the HD2A Program was the bringing together of a group of independent projects based on a common goal of using data in innovative ways to drive clinical and administrative decisions related to prevention and treatment of opioid use disorders and/or pain management. Each project had a distinctive set of aims and activities and worked with different target populations and sources of information in different clinical settings. However, projects have been brought together in two in-person meetings and quarterly learning collaborative sessions to share information, exchange ideas, and learn from one another’s experience in translating data to action.

The third distinctive feature of the HD2A Program was the creation of three Support and Resource Centers to provide technical assistance and related resources in key components of the translation of data to action: implementation, modeling and economics, and data acquisition and management. The Research Adoption Support Center (RASC) provides consultation on adoption and implementation of evidence-based practices, along with a coordination and communication infrastructure for the whole program (7). The Modeling and Economic Resource Center (MERC) offers consultation on economic analysis, simulation modeling, and behavioral economics to estimate impact and cost-effectiveness (8). The Data Infrastructure Support Center (DISC) provides data support, tools, training, and modernization, and serves as a connection point to the HEAL Data Ecosystem (9). The DISC also received funding to provide Rapid Data Infrastructure Modernization Support (Rapid DIMS) projects, which are small supplements that the Innovation and Acceleration projects could apply for to obtain datasets or other data-related needs. In addition, the RASC is designed to enhance the potential for translation and sustainment by integrating expertise in implementation science methods, the MERC to integrate economic elements to support impact and eventual scalability and sustainment, and the DISC to coordinate approaches to common or harmonizable data elements.

The final distinctive feature of the HD2A Program was the strong encouragement by NIDA that investigators involved in human-subjects studies employ a common set of tools and resources that will promote the collection of comparable data across studies and to do so by incorporating the measures from the Core and Specialty collections.

Many of these features are characteristic of other NIH initiatives, including the NIDA-funded Justice Community Opioid Innovation Network (JCOIN) (10), Juvenile Justice-Translational Research on Interventions for Adolescents in the Legal System (JJ-TRIALS) (11), and Integrative Management of chronic Pain and OUD for Whole Recovery (IMPOWR) (12), and the National Cancer Institute-funded Population-based Research to Optimize the Screening Process (PROSPR) (13), Breast Cancer and the Environment Research Program (BCERP) (14), and Cohorts for Environmental Exposures and Cancer Risk (CEECR) (15). These initiatives are designed to link similar projects organized by a common theme. The goal is to create opportunities and impact that go significantly beyond the capability of independently conducted research projects. Purported mechanisms include shared learning, collaborations, training and mentoring, and data pooling. The envisioned total would be greater than the sum of the parts (12).

However, the extent to which such initiatives help to accomplish NIH goals and objectives remains unclear. Also unclear is the extent to which these features, separately or collectively, provide added value to the efforts of individual projects. To address these questions, we have been conducting a formative evaluation of the performance of the research projects funded by NIDA as part of the HD2A Program. As part of the mixed methods evaluation, we conducted semi-structured interviews with project grantees and members of the three support networks to assess the following: 1) the progress being made by individual projects in achieving their project aims; 2) potential obstacles to achieving these aims; 3) types and extent of support provided to projects by research support centers and support mechanisms; 4) needs for additional support; and 5) overall assessment of participation in the HD2A.

## METHODS

Authors followed the Standards for Reporting Qualitative Research (SRQR) (16) to report the findings from this study (see Supplementary Materials).

### Participants

As part of the formative evaluation of the HD2A Program, semi-structured interviews were conducted beginning in March 2023 with 15 representatives of 6 NIDA-funded projects targeting prevention and treatment of opioid use disorders and improving pain management. These projects had been in operation for approximately six months at the time of data collection and constituted the first of two HD2A cohorts. In March 2024, the six Cohort One projects were joined by 4 new projects, referred to as Cohort Two, in a second series of semi-structured interviews with 24 project PIs and key personnel to assess progress made by all 10 projects since their funding began (18 months for Cohort One and 6 months for Cohort Two). In March 2025, a third round of semi-structured interviews was conducted with 33 project PIs and key personnel of all projects in both cohorts to assess their progress over the previous year. In addition, interviews were conducted with 8 members of the three HD2A support centers in January 2025. The study protocol was reviewed and approved by the Weill Cornell Medicine Institutional Review Board.

### Data collection

All interviews were conducted with two or more members of each project team on the Zoom platform and were recorded after obtaining verbal agreement. The first author, a medical anthropologist with several years of experience in conducting qualitative research, began each interview by confirming agreement to record the interviews. Each interview lasted between 50 and 60 minutes and utilized an interview guide. In the 2023 interviews, participants were asked to describe their project aims and report on progress made since the start date of the award. Specific questions were then asked regarding successes and challenges experienced by the project to date, interactions with the three research support centers, and overall experience with the HD2A Program. In the 2024 and 2025 interviews, specific questions also included success in implementing the intervention they were funded to develop or scale up, whether investigators made any pivots to achieve project aims, success in getting community buy-in for implementation efforts, and participation in and assessment of learning collaborative sessions. In the 2025 interviews, participants were also asked about involvement in efforts to harmonize the data being collected by each project.

Prior to terminating the interview, the information provided by the participants was summarized by the interviewer for the purpose of confirmation (validation) and creating an opportunity for additional information to be provided by participants. The first author then prepared a summary of each interview and reviewed and edited the interview transcripts provided by Zoom.

### Data analysis

Interview transcripts were analyzed using a thematic content analysis methodology (17). First, transcripts were reviewed individually by two investigators (LAP, EL) to develop a broad understanding of content related to the project’s aims and to identify topics for discussion and observation. Short descriptive statements or “memos” were prepared to document initial impressions of topics and themes and their relationships, and to define the boundaries of specific codes (i.e., the inclusion and exclusion criteria for assigning a specific code) (18). Second, transcripts then were coded to condense the data into analyzable units. Segments of text were assigned codes based on a priori (i.e., from the interview guide) or emergent themes. Text segments relating to barriers and facilitators of individual project progress were coded according to the five domains of the Consolidated Framework for Implementation Research (CFIR)(19): characteristics of the intervention being implemented (e.g., cost, adaptability); characteristics of the outer domain (i.e., external environment); characteristics of the inner domain (i.e., organizations where implementation is taking place); characteristics of the individuals involved in implementation (i.e., patients and providers); and characteristics of the implementation process (e.g., planning, leadership, monitoring and feedback). Codes were also assigned to describe connections between categories and between categories and subcategories (18). Lists of codes developed by each investigator were matched and integrated into a single codebook. Third, one transcript was independently coded by two members of the research team. Disagreements in assignment or description of codes were resolved through discussion between investigators and by refining definitions of codes. With the final coding structure, two investigators each separately coded randomly selected samples of three transcripts and reached a level of agreement of 94% for primary codes and 65% for secondary codes. Coding discrepancies were resolved through consensus and the codebook was modified and transcript codes revised accordingly. Fourth, a hierarchical series of categories was generated using Dedoose (20), a cloud-based data management program. Fifth, the process of constant comparison was used to further condense the different categories into broad themes.

## RESULTS

Data from the interview transcripts were grouped into three overall themes with several subthemes: 1) accomplishments and challenges of individual projects; 2) HD2A Support Center activities; and 3) overall assessment of participation in the HD2A Program. Each of these themes and their respective subthemes are described in detail below, along with tables containing illustrative quotes.

### Individual project accomplishments and challenges

#### Project aims and activities

All research projects funded under the HD2A initiative have the goal of reducing opioid overdose, improving treatment of opioid use disorders, or improving pain management by using data obtained from sources such as individual patient Electronic Health records (EHRs) and/or large administrative data sets like Medicaid claims data to guide provider decisions regarding patient treatment and recovery. The projects span a range of health care settings (Emergency Departments, inpatient care, primary care, and specialty medical care) and service systems (e.g., public health). During the exploratory R61 phase, project teams worked to get input from stakeholders on relevant data for decision-making, implementation strategies and desired support tools; gain access to individual patient data and administrative data sets; developed data dashboards and other electronic tools for providers; finalized intervention and research protocols for the phase 2 trial; and recruited healthcare systems and providers for participation in phase two activities. In the second phase, investigators are or will be conducting an RCT or other rigorously designed study to evaluate whether the data use interventions achieve intended aims of improving treatment and implementation outcomes. Quotes from project participants illustrating performance to date are presented in Table 1.

### Accomplishments

In the last three years, all projects reported having made some progress in achieving their aims. In year 3, seven of the ten projects had already successfully transitioned from the R61 exploratory phase to the R33 confirmatory phase; the remaining 3 projects were on target for submitting their R33 applications. (As of this date all have received R33 funding.) Most of the challenges reported in previous years were successfully addressed due to information and advice provided by the HD2A Support Centers and other colleagues, changes in the inner and outer setting determinants of implementation, or modification of implementation strategies. These changes enabled projects to overcome challenges associated with data access and visualization, stakeholder engagement and patient participation in project activities.

Among the accomplishments cited by representatives from each of the projects were the following: construction and implementation of an electronic dashboard for data visualization; demonstrations of intervention implementation feasibility; successful engagement with and recruitment of community partners; mini-pivots to improve the intervention or overcome barriers encountered in its implementation; a “hard-pivot” to redesign the implementation strategy; and submission of manuscripts for publication. Project representatives reported being well-prepared to transition into the R33 phase of their projects and learn how to implement their intervention, measure outcomes, and disseminate information necessary for intervention scale-up.

### Challenges

Despite the overall record of success to date, each project has encountered several challenges in achieving their aims. One of these challenges involved assessment of implementation processes and selection of appropriate strategies for implementation of the various programs and practices being developed and evaluated. This challenge was successfully addressed through assistance from RASC core faculty and development of collaborations with other implementation research projects to acquire expertise in implementation science to assist in their efforts to implement their planned interventions. A second challenge experienced by the research projects had to do with securing the engagement of community partners. Often, this occurred as a result of changes in the CFIR outer setting (19) specifically the shift in context resulting from the recent epidemic of fentanyl overdoses and shortage of healthcare providers. After two or three years of operation, some of these barriers remained for some projects. On the other hand, several changes in the outer setting were noted by interviewees that have facilitated the implementation of their interventions and achievement of their project aims.

Another challenge was related to access to administrative/EHR data necessary for intervention development. One project was able to secure a memorandum of agreement from a state Department of Health, giving them access to statewide data on overdose fatalities. With the assistance of additional funding from NIDA, another project acquired access to a pharmaceutical sales data base, enabling them to chart suboxone prescriptions. A third project now has access to an integrated data set managed by a county Community Health Services department. Access to data sets such as these has led to progress in dashboard development and linking provider prescribing data with patient outcomes data.

Although some challenges have persisted, the projects have managed to develop alternative strategies for implementing their interventions. For instance, after a proposed partnership failed to materialize, one of the projects entered into a partnership with other providers, supported, in part, by the passage of a countywide initiative for the expenditure of $1 billion for community care centers. To address the challenge of promoting the adoption of an innovative treatment strategy by providers that are resistant to change, one project trained implementation facilitators in motivational interviewing and greater engagement with providers in development of materials and the intervention. After their pilot training experience with one of their partners, investigators of another project decided it would be better to make it more directive and to conduct follow-up using monthly technical assistance calls.

New challenges encountered in the subsequent years included developing and implementing a data dashboard in the context of staff turnover and a dynamic clinical environment; training partner staff in the use of the dashboard; enrolling study participants; obtaining training in community-engaged research; the desire of health system partners to roll out the intervention before it is properly evaluated and successfully implemented, necessitating a change in research design; and securing participation from geographically dispersed, predominately non-academic clinic sites.

In Year 3, three of the projects experienced delays in meeting milestones, necessitating in two cases a decision to apply for a no cost extension (NCE). These delays included participant enrollment due to clinic understaffing, completion of clinic construction, or patient reluctance to participate. A few of the project representatives also reported ongoing issues with engaging community partners (clinics, state agencies) due to lack of trust and staffing turnover. New challenges encountered during the past year included difficulties accessing or merging patient data (4 projects) and a lower than anticipated rate of intervention adoption by providers (2 projects) or patients (2 projects). Representatives of other projects reported no major challenges encountered in the past year.

### Need for support

Projects that were still early in the R61 phase when interviewed reported that they needed more time to assess needs for support moving forward. Representatives of three projects reported that the need for support was anticipated to occur more in the R33 phase than in the R61 phase. Representatives of other projects reported needing assistance with planning for implementation, scale-up and sustainability of interventions; gaining access to patient EHR data; and data analysis. Two projects reported seeking assistance in submitting requests for Rapid DIMS funding for project-specific needs such as creating an inventory of local data sources and more systematic policy surveillance or hiring a postdoctoral fellow to assess implementation process or performing cost analyses.

### HD2A Support Center activities

Support was available to individual projects in three different forms, tailored support for individual projects obtained from periodic check-ins by support center leadership, Rapid DIMS projects that funded individual projects or groups of projects, and participation in monthly learning collaborative sessions. Each form of support is described below.

### Tailored support for individual projects

There are three venues by which the HD2A Support Centers assist grantees in completion of their project aims (Table 2). The first venue is through interactions that are initiated by the grantees themselves. In the past year, these interactions revolved around specific project needs, including dashboard development, community engagement, data access, funding for Rapid DIMS projects, and planning for implementation, scale-up, and sustainment of interventions. The second venue is through interactions initiated by the support centers. These include periodic check-ins, presentations at the in-person meeting, assessing implementation readiness and progress through the application of standardized tools, and hosting learning collaborative sessions. The third venue is through interactions based on longstanding professional relationships between individual project team members and support center technical experts. Some investigators were familiar with RASC technical experts through their involvement in the Implementation Research Institute based at Washington University in St. Louis. Other investigators served in dual roles as project investigators and support center technical experts, allowing for a “seamless transition” from one role to the next.

Of the three support centers, perhaps the most engagement by grantees was with the Research Adoption Support Center (RASC). RASC has three aims: 1) to serve the innovation and acceleration projects by providing technical assistance on demand, especially during the second phase (R33) of their grants; 2) to be a national resource for NIDA-funded projects to help them infuse implementation, science methods and identify pragmatic resources like the Implementation Research Logic Model (IRLM) (21), and the Dissemination and Implementation Research Capability- Self Survey (DIRC-SS) (22), a collaborative decision-making tool for implementation planning; and 3) provide shared infrastructure to enhance the coordination and communication across the HD2A Program, including the research projects, support centers, and NIDA. Support provided by the RASC team to individual grantees in the past year included evaluation of capacity and progress toward implementation by completing the Inventory of Factors Affecting Successful implementation and Sustainment (IFASIS) (23) and DIRC-SS tools, assistance with designing process evaluation, feedback on dashboard development, collaboration on manuscripts, and assistance with intervention implementation. However, the RASC team reported that adoption of measures developed by the center and recommendations for implementation has been slow and inconsistent among the projects. For their part, representatives from six of the ten projects commented on the time and effort involved in attending meetings hosted by RASC and completion of the implementation assessment tools; in a few instances, there were reports that such requests were time-consuming and interfered with completion of other project-specific activities, particularly those in the R61 stage.

The role of the Modeling and Economic Resource Center (MERC) is twofold: 1) a service-oriented one in which center technical experts consult with HD2A grantees; and 2) conducting their own research on reimbursement models that have value to the initiative and the field as a whole. Center directors reported greater success in fulfilling the second role than the first one. One of their chief accomplishments in Year 3 was the development of an interactive tool for modeling that forecasts how payments are covered in collaboration with one of the projects in which a MERC member is also a co-investigator. Other projects are focused on adaptation of simulation modeling for use online and looking at how micro-costing results can inform sustainability, transportability, feasibility, and generalizability. With respect to consultation, grantees were classified by the MERC team into three groups. In the first group, projects that had health economists on the team. MERC had limited engagement with these projects because they already had many of the resources they needed. In the second group of projects, grantees indicated that they were going to do cost effectiveness analyses but devoted much of their time during the R61 phase to planning and getting to the next phase. In the third group of projects, there was no real need for cost effectiveness support by nature of their aims (e.g., working with a large data set that project investigators were already familiar with).

For its part, the DISC has assisted projects seeking funding for specific activities such as access to data sets, as well as consultation on the development of data infrastructure, adherence to new NIH requirements regarding data sharing and storage plans, dashboard visualization development, and development of data collection instruments. DISC technical experts have developed tools to support data collection and management for each project and maintains contact with each project through periodic check-ins to address any challenges they may be experiencing with respect to access to and use of data to simultaneously meet their own specific aims and NIH expectations of data harmonization across projects.

### Rapid DIMS projects

Administered by the DISC, the Rapid DIMS program provides funding for activities that address gaps in knowledge or other barriers to using data for action. The Rapid DIMS supports data modernization at the interface of chronic pain and opioid use disorder to advance research in important areas of focus: data infrastructure, data acquisition, data visualization, data collaborations, or related data activities. In 2023, five projects were awarded Rapid DIMS funds, two projects to apply social network modeling within each project to learn more about how key system players share and encourage the uptake of harm reduction principles among other stakeholders across their networks; one project to expand testing and integration of a new algorithm into electronic medical records (EMR) making it accessible to clinicians in real-time; one project to purchase data from the IQVIA dataset, support efforts to curate a state-wide report of opioid-involved accidental or undetermined fatal drug overdoses, and develop and maintain a QR code-based enrollment platform; and one project to support the integration of patient-reported outcomes (PROs) into the EHR to complement a clinical decision support (CDS) to promote 2022 CDC guideline recommended practices. In 2024, only one project was awarded DIMS funding to support select stakeholders, develop geospatial tagging within a QR-based platform, and automate the project’s enrollment process.

### Learning collaborative sessions

Quarterly, representatives from each of the projects are invited to participate in a learning collaborative session hosted by the RASC. The aims of the sessions are to present information on topics of general interest to the project research teams and provide opportunities for teams to report progress and discuss challenges in achieving their respective aims. Members of all three support centers have given presentations at learning sessions in the past year. RASC representatives presented on the IRLM; MERC representatives presented on the revenue tool they had developed in collaboration with a grantee; and DISC representatives presented on data visualization and use of Medicaid data. Presentations have also been given by project representatives.

### Overall assessment of participation in the HD2A

To date, investigators from most of the teams have reported the experience of participating in the HD2A Program to be positive, citing the opportunity to interact with other investigators and obtain assistance from the support centers as particularly beneficial, as illustrated in Table 3. For some investigators, the assessment was more nuanced, having described their experience to date as more mixed.

### Assessment of support provided by centers

The number of projects that reported support specific to each center by year of funding is indicated in Table 4. Projects were assigned the classification of “helpful” if representatives reported a specific benefit such as obtaining access to a particular dataset or guidance to measuring process or outcomes, a specific opportunity such as participating in and publishing a paper related to their project aims and the center goals, and/or commented on how helpful the support center had been. Projects were assigned the classification of “To Be Determined” or TBD if they were either unfamiliar with the scope of services offered by each center or were anticipating a need for a particular form of support at some point in the future. Projects were assigned the classification of “Not needed” if they either expressed dissatisfaction with the requirements for receiving the support (e.g., having to complete assessments or questionnaires that appeared to be unrelated to the aim of their project) or reported no need for the services offered.

The most detailed comments provided during the project team interviews pertained to interactions with the RASC with each of the projects providing comments. Most of the comments were positive in all three years with 61.5% of projects finding the support provided by RASC as a whole or specific RASC investigators to be “helpful”, “very helpful” or “super helpful.” In the first two years of their funding, there were a few projects that either anticipated a need for RASC assistance in subsequent years or were unsure as to what kind of support RASC could provide them. One investigator each in Years 2 and 3 indicated no need for support from RASC, and in Year 3, two of the first cohort projects pointed to the burden associated with completing tasks such as the DIRC-SS questionnaire. Half of the projects also reported support from DISC to be helpful, while half of the projects reported support from MERC to be determined.

#### Assessment of Rapid *DIMS funding*

In their first year of funding, most projects were unaware of the availability of funds to support Rapid DIMS projects to address specific needs or felt that they had little time to focus on applying for such funds. For those who did receive such funding, however, comments were generally quite positive (See also Table 3).

### Assessment of learning collaborative sessions

All projects have participated in most of the HD2A learning collaborative sessions and reported that their chief benefit has been learning about what the other projects are doing. Some project teams also cited the benefits of sessions that covered specific topics such as dashboard development and implementation planning. Being asked to lead a learning collaborative discussion was also cited as a positive feature of the sessions.

However, representatives of five of the projects were of the opinion that the learning collaboratives were not engaging in collective problem solving or assisting in helping projects address their specific problems. This was attributed to the perception that their own project was “unique” and stood out from the other projects (e.g., having a focus on specialty versus primary care or clinical versus community). The absence of a common set of problems faced by the projects was also cited by RASC leadership as a reason why the sessions did not adhere to the traditional model of learning collaborative operation.

### Assessment of R61/R33 mechanism

Some of the interview participants expressed confusion concerning the transition from the R61 phase to the R33 phase of their projects. One of the MERC technical experts also commented on the challenges posed by the R61/R33 mechanism, noting that grantees tended to be preoccupied with the transition to the R33 during the R61 stage. Nevertheless, representatives of individual projects all commented favorably on the feedback and assistance provided by NIDA program officers when preparing applications for the transition from the R61 phase to the R33 phase of their projects.

### Interactions with other projects

With respect to the interaction with other projects, opinions of the individuals interviewed were decidedly mixed. On the one hand, as noted earlier, project investigators cited the opportunity to engage with and learn about the other projects as one of the positive features of the HD2A program. Some of those interviewed pointed to specific instances of collaboration with other projects on specific studies such as the assessment of community partner social networks or dashboard development, while other grantees felt that learning about the activities of other projects helped them to place their own project within the broader context of the goals of the HD2A Program. Interactions with other grantees during the in-person meeting and learning collaborative sessions also provided opportunities to network and identify potential areas for future collaboration.

Nevertheless, representatives of three of the projects expressed the view that the value of bringing together such a diverse set of projects was limited because each project viewed themselves as standing apart from the others in one way or another, even when more than one project had certain elements or activities in common, such as dashboard development. This view was shared by members of the RASC and MERC support centers. Further, as noted earlier, six of the ten projects commented on the time and effort associated with engaging with the support centers in the form of more meetings and more requests to provide information as part of evaluation and capacity assessment efforts.

### Data harmonization

With respect to the HD2A goal of promoting data harmonization, representatives from each of the projects commented that they could find little evidence of significant developments toward achieving that goal, which was attributed to the fact that plans for data collection had been developed by each project prior to their awards with little guidance on what data should or could be harmonized across the projects.

## DISCUSSION

The support provided to projects funded through the HD2A Program includes several unique features that evolved out of prior NIDA-funded experiences with center support mechanisms (10, 11), including the three-support center model designed to provide specialized and expended services in data infrastructure, implementation science, modeling, and economic services. These centers provide grantees with direct interactions with national experts so that their unique needs can be met while setting the stage for sharing knowledge and data through harmonization. This is also the first initiative that capitalizes on the R61/R33 mechanism. During the R61 phase, in addition to revising and improving their research strategies based on contextual realities, in this preparatory phase, projects are presumably evolving and receptive to adopt methods and measures not proposed in their original applications for the R33 stage. As a major part of a formative evaluation, conducted half-way through the project, semi-structured interviews were intended to provide feedback to the centers, research sites, and NIDA regarding progress during the research projects R61 phase and examine opportunities for adjusting efforts as the projects transition to the R33 phase. However, the information gathered from these interviews also provides some valuable lessons for the design and execution of future initiatives intended to bring together and support a diverse array of approaches to addressing a significant public health issue.

As is the case with any research project funded by the National Institutes of Health, the projects participating the HD2A Program have experienced their share of success and challenges. To date, every project has successfully transitioned from the exploratory R61 phase to the confirmatory R33 phase with the assistance of guidance and support provided by NIDA, HD2A research support centers, and their own professional resources and networks. A few projects encountered some unanticipated delays in making this transition, necessitating requests or consideration of a request for a No Cost Extension. All projects have nevertheless experienced challenges of one form or another in achieving their aims, including administrative delays in securing IRB approval and memoranda of understanding (MOUs) with community partners; development of partnerships with providers, health care systems and state agencies; obtaining access to clinical and administrative data; and implementation of their proposed interventions.

Challenges such as these are not uncommon among NIH grantees regardless of funding mechanism. What distinguishes this particular group of grantees, however, is their experience with the four distinctive features of the HD2A Program: the R61/R33 mechanism, the involvement of independent projects based on the common goal of using data in innovative ways to drive clinical and administrative decision-making, the availability of technical assistance and related resources through three research support centers, and the encouragement to participate in the harmonization of data collected by each project. For some of the HD2A grantees, the lack of familiarity with the R61/R33 mechanism posed a challenge, leading to confusion and at times frustration over the process and requirements for transitioning from the exploratory R61 phase to the confirmatory R33 phase of their proposed projects. However, this challenge was successfully addressed through interactions with NIDA personnel.

Attempts to bring the projects together through an in-person meeting and learning collaborative sessions were viewed positively by all projects, although representatives from some of the projects viewed the potential benefits of these activities as being limited by unique characteristics of their own projects. This may also account for the fact the specific collaborations between and among HD2A projects were relatively few and limited in scope. An earlier study of National Cancer Institute-funded research consortia found that groups of investigators that are independent at the outset but are required to collaborate have less overlap in scientific scope, design, aims and methods, making it more challenging to form cross-consortium collaborative research than groups that submit a single grant application with a cohesive set of aims and projects (24).

Each project acknowledged the value of having access to the talent and expertise of each of the three support centers, especially in implementation science. This is not surprising, as several mapping reviews have identified the low proportion of NIH grants that identify any implementation theory or measure to be collected (25–27), and it supports the value of having a center with this expertise available. It is also a cost-effective way to infuse state of the art implementation science. Some projects stated that access to support should have been available when they were preparing their applications for submission to the HD2A initiative rather than afterward. That support would have enabled them to better prepare for such assistance and align it with the needs of their specific project. Similarly, it was not entirely clear if the leadership of the three centers were aware of those needs when they submitted their applications for grant support. Moving forward, greater engagement between those providing technical assistance and those receiving it should occur earlier in the process of designing and implementing projects. Perhaps the RFAs for the centers should go out before the RFAs for individual grants. Potential applicants could then consult with support centers prior to submission of their applications. RFAs should also be more specific on anticipated common data elements.

Projects that had secured funding or otherwise participated in a Rapid DIMS project uniformly expressed appreciation and gratitude for the resources and opportunities available from this mechanism. Rapid DIMS funding enabled them to address important questions or acquire access to data such as the IQVIA health claims data that benefitted other HD2A projects in addition to their own. The DIMS funding represents perhaps the most impactful form of support provided by the HD2A Initiative. However, many projects reported having little understanding of the usefulness of the funding, and some reported they had insufficient time to submit an application or execute the project due to other demands placed upon them in achieving their overall project aims. A few project leaders also reported being unable to use this mechanism to support hiring of postdocs or other researchers to carry out certain tasks related to data sharing or economic modeling. It is recommended that more information be provided regarding the purpose and potential of DIMS awards to funded projects in NIH Program Announcements or Notices of Funding Availability, and that results of projects supported by this mechanism be disseminated to other projects.

For the most part, project investigators were satisfied with their participation in the learning collaborative sessions, especially those that were focused on areas that were particularly relevant to project aims such as dashboard development and community engagement. However, all of the project representatives interviewed commented that the primary benefit gained from participation was learning about what other projects were doing. Some pointed to the networking opportunities afforded by learning collaborative participation that could lead to future collaborations. Nevertheless, the utilization of plan-do-study-act cycles or collective problem solving to address issues common to the projects that are a fundamental feature of the learning collaborative model (28, 29) were not features of these sessions. This was attributed to the great diversity in project aims and approaches as each project described themselves as being unlike the others. This raises the question of whether the learning about other projects requires quarterly attendance of learning collaborative sessions or could be achieved by some other means. It also raises the question of the value of attendance when there is so much diversity in project aims and approach. It is recommended that learning collaborative sessions should devote more effort to engaging participants in collective problem solving.

Finally, there was consensus among project investigators that efforts to harmonize the data being collected by each project were not progressing as anticipated, and that this could be attributed to the lack of clarity as to whether or not this was a funding requirement or rather a suggestion, as well as the specific and at times restrictive nature of the data being collected to achieve project aims. Previous research has identified numerous challenges to harmonizing data from NIH collaborative network initiative participants (30). It is recommended that NIH institutes and centers be more prescriptive in their expectations of data harmonization when soliciting requests for applications.

### Limitations

Recruitment of participants with different roles within different types of projects across the U.S. enabled us to acquire varying perspectives on the benefits and challenges experienced as participants in the HD2A Program, including the perspectives of those providing support and those in need of support. The rigorous collection and analysis of the data, including summarization of information obtained during the interview by the interviewer and the review of interview summaries by project investigators, supports the internal validity and reliability of findings. There are, nevertheless, several limitations to this study. This study was designed as part of a formative evaluation of progress being made by a relatively small number of individual projects in achieving their specific aims and the extent to which participation in the HD2A Program contributed to that progress. The data used to conduct this evaluation is based entirely on self-report with more attention given to project process than outcomes. Research to validate the findings of this study and assess the impact of HD2A participation on project productivity is ongoing. Further, this paper reports on evaluations conducted in the first two years of operation of both cohorts and the third year of operation of Cohort 1 only. Challenges encountered in meeting project aims and extent and impact of support provided is likely to change as projects fully transition into the confirmatory R33 phase. At that point, more objective metrics like number of publications and intervention and implementation outcomes will become more available and more relevant to the aims of this evaluation.

## CONCLUSION

Despite these limitations, formative evaluations to date have found substantial evidence that the HD2A Program has made considerable progress toward its goal of making data more real-time and useful to accelerate the implementation of evidence-based interventions for opioid use disorder and pain management with some notable exceptions. Despite being viewed by some investigators as burdensome and a distraction from more immediate needs, all projects have obtained needed guidance and support from the three research support centers, particularly through supplemental funding to support rapid data infrastructure projects. Interactions with other projects at an in-person meeting and learning collaborative sessions are viewed quite favorably, even if such interactions provide limited opportunities for research collaboration and collective problem-solving due to unique features of each project. Transitioning from the exploratory R61 stage to the confirmatory R33 stage proved to be a minor challenge for some of the projects, one that appears to have been successfully addressed with the assistance of NIDA personnel. Efforts to harmonize data are challening and will require additional support and guidance. Future research on the experience of participation in other NIH-funded research consortia is recommended to determine which findings reported here are unique to the HD2A Network and which are common to efforts to build and support other research networks.

## Supplementary Material

Supplementary Files

This is a list of supplementary files associated with this preprint. Click to download.

• BMCOpenSRQR103025.docx

## Figures and Tables

**Table 1:** Individual project performance subthemes

Subtheme	Illustrative Quote
Accomplishments	*We’re in a great place now, just being able to identify those patients. And I guess the first two years is building the infrastructure to identify the patients, identify the processes and the different pathways. And again, the people that are involved in that process, I think a lot of that just needs to be built out. And so that’s what we’ve been documenting, exploring so that once we have more patients coming through, that dashboard will update us not in real time, but every 24 hours, we will be able to use those data to see where we’re at and I think more systematically be able to implement some changes and improvements and see how that affects our outcomes. (Cohort 1, Project 2, Year 2)*
Challenges	
Outer setting	*Our health data warehouse has long… been a challenge for our system. I think they set it up with the intention of it being free and having to facilitate both clinical as well as research. It’s been around for a while. They’ve had a couple of different directors in, and it’s just never really hit. And one of the things we actually use is the insurance data. They’ll pay our claims data on a prior project and after lots of time, money and investment. The output was pretty useless, and we ended up not actually being able to use it. (Cohort 1, Project 4, Year3).*
Inner setting	*It’s just an inherent challenge in this work. It’s an exploratory and sort of iterative group model building process for the data products and data linkage prototype approaches that we’ll be designing and testing for this phase or use in this hopefully funded second phase. And because of that iterative and exploratory process, it does take a lot of reminding people what our goals are and what we’re doing. Because people are busy right? They’re not just waiting around to come to our stuff. They’re moving and shaking stuff in the said landscape in [state] all the time. And they don’t want this to just be another meeting they go to, and we don’t want that either. So, one of the challenges we have is really framing the goals of this project while letting them know that they’re in charge of shaping the goals, too. Yeah, it’s a little bit like an intricate dance that we do together with our essential community partners. (Cohort 2, Project 3 Year 1)*
Addressing challenges	*So, I guess the biggest thing is in consultation with [RASC team members]. We thought about going to a ROlO [Roll Out Implementation Optimization] rather than kind of a traditional stepped wedge. So, we got together with [RASC technical expert]. We actually had him come out for a half day training on alternative stepped wedge design… So that is something that we’re planning on, which seems to fit more because we thought we’d roll out the dashboard with each successive cohort. But instead, the State said, ‘Hey, we got to make this public for everyone.’ Great! So, it’s public now. So now, instead, we’re kind of rolling out the training and technical assistance. And the idea is we’re going to be shifting that training, technical assistance based on monthly feedback and reports and on fidelity monitoring. So that is one thing that came out of consultation with the RASC. (Cohort 1, Project 6, Year 2)*
Need for support	*I feel like it might make sense to think ahead about the implementation phase because I feel that there is such great variation across the [sites]. So even if we develop a single package of innovations, how they are delivered to these different [sites]s based on their needs and the specific issues that that are affecting them. I feel like it’s something that maybe the RASC could help us think about a plan ahead because… there are quite entrenched practices, cultures, leadership issues that there may be resistant to any type of intervention. (Cohort 1, project 1, Year 1)*

**Table 2:** Types of assistance provided by the support centers subthemes

Subtheme	Illustrative quote
Initiated by grantees	*And we met with [MERC technical expert] this morning. We’ve been meeting regularly about how we can use these data in terms of modeling and kind of take it to the next level to kind of make the case that ties a little bit into that cost effectiveness as well. So, we have a couple of those demo grants that supported these different kinds of extra level activities. (Cohort 1, Project 2, Year 2)*
Initiated by support centers	*And we’ve been working a lot with RASC. In fact, we were just talking about getting back in touch with [RASC technical expert], and he keeps thinking that the implementation science lessons we’ve learned in doing the work for the R61 is a really good implementation science learning lesson to write up, and we’re in the process of working, figuring out how to work with him to write this up in in a way that is useful for people who are doing similar work. He thinks it’s a terrific idea that we do this. And we’re far less knowledgeable about implementation science phraseology, even the literature that’s there. And so, we’re hoping to work with him as either a co-author or as a consultant, as we try to put all that we’ve learned into an IS [Implementation Science] framework. (Cohort 1, Project 1, Year 3)*
Longstanding professional relationships	*We touch base with the RASC fairly often. We just see [RASC technical experts] at conferences. And now [project investigator] is a part of the training program that [RASC technical expert] has. So that’s like more facetime. So, we’ve shifted with them... I’m a big fan of [RASC technical experts] and everyone so they’re all good buddies, all good friends. The consultation has been helpful... So, we’ve interacted a lot with the RASC. There’s some natural connections like I saw [RASC technical expert] at the D&I [Dissemination and Implementation] meeting. So, I think there’s just some natural connections that we already have with them. (Cohort 1, Project 6, Year 3)*

**Table 3:** Assessment of HD2A subthemes

Subtheme	Illustrative quote
Positive overall assessment	*I think the most tangible aspect of that has been the way that the RASC has encouraged us to apply a little bit more of an implementation lens and to help us think about the work that we should/we could be doing now. That would be consistent with that later on, even though that is not necessarily a strong focus of our project, but would be something that would be interesting to apply and help to develop. (Cohort 2, Project 4, Year 2)*
Mixed overall assessment	*I think it’s mixed from my perspective. There are parts of it where I do appreciate the connection with colleagues and folks that we come in contact with. But I do feel like there’s like a little bit of a burden from having the RASC and the DISC and the MERC, all wanting to do things with us. Right? I understand their incentives to do that, but they are creating in some ways more work not necessarily facilitating our work, if that makes sense. (Cohort 2, Project 2, Year 2)*
Assessment of support provided by centers	
Helpful	*We’ve been working with the DISC around a rapid DIMS to promote a study and then also some work to help us make sure that we are staying compliant and consistent with the other requirements of the HEAL network generally. So, we really appreciated the DISC’s support in that work. And we’re going to continue working with them through that, being very well supported and very confident, then, the things they’re going to be able to offer will be of help to us. (Cohort 2, Project 4, Year 2)*
To be determined	*And then, the MERC. I think they were suggesting doing some type of economic evaluation as well. But that hasnť even come up so a little bit of lack of clarity as to in whose court does it fall? But I imagine when we get to the transition point from the R61 to the R33, that we’ll start to accelerate some of those discussions with them. (Cohort 2, Project 2, Year 2)*
Not needed	*We haven’t interacted with the work or the MERC as much, partly because we don’t have a cost component to our work. We would have loved to, but we just didnť have the budget for it. And we’re not doing much in the simulation model side of things because the data is owned by the State. And we’re still working into getting access to that system, much less sharing it with someone outside of the State. (Cohort 1, Project 6, Year 1)*
Burdensome	*I mean, the RASC has their implementation science measure that is this multipage tool that they always want us to fill out. And you know we all love [RASC expert], and personally I like [them] greatly right. But the use of those tools is just this added burden, right?... But this is true of a lot of things where we have coordinating centers. I mean, I see this in our in the Clinical Trials Network, our coordinating center. There’s just huge frustration with them because they do create a lot more work for everybody. So, I would say in the universe of things, HD2A is doing pretty well because for the most part they just let us do our work. But it does come up now and then. (Cohort 2, Project 2, Year 2)*
Assessment of DIMS funding	
Lack of time or awareness	*I was lamenting that we didn’t get to apply in that last rapid [DIMS project opportunity]. Part of it was, we got a little delayed just by sort of other deadlines, but also there’s this confusion for us at first about whether if we were awarded one, how we would deal with that. Given that we were in this phase with the plan,*
	*we’re hoping to be on time and finish it, and we couldn’t finish the rapid supplemental project that quickly. (Cohort 2, Project 1, Year 1)*
Positive experience	*We asked very early on in this process for funds to get pharmacy sales, data for drugs used to treat opiate use disorder most notably suboxone, buprenorphine and that to get that data you can. There’s one source called IQVIA, which is basically pharmacy data that’s compiled by... a commercial company, and they charge an arm and a leg to corporate entities who want the data. Well, they’ll also charge an arm and a leg to researchers. There’s no such thing as an academic discount that I’m aware of. But we actually got NIDA to pay for it through the through the DIMS, I believe, and we just got a note from them that, as of fifteenth of March, we have a scope of work agreement that says we’re going to get the data we want. (Cohort 1, Project 1, Year 2)*
Assessment of Learning Collaborative Sessions	
Helpful	*I think there was one that was about contextual determinants and sort of approaches to interviews that was really helpful. Yeah, yes, helpful on the implementation science front for sure. (Cohort 1, Project 5, Year 3)*
Not helpful	*We’re somewhat unusual in that our efforts are more at the community level outside of the clinic than health care. So, sometimes, finding commonalities, they’re not readily apparent, and the learning collaboratives... the one I attended seemed to be structured in a way that the topic that’s decided upon drives the content rather than the needs of the projects drive the discussion. (Cohort 1, Project 1, Year 2)*
Assessment of R66/R33 funding mechanisms	
Confusion concerning transition	*So, we have a 2year, R61 phase. And then we’re getting ready to submit our documents, which has been a super vague, weird process. We still don’t exactly know the documents we’re supposed to send... Do we have to resubmit everything, and highlight changes, and then have a cover document talking about how we met the milestones. And so, anyway, yeah, we’re close to wrapping up year 2. So, getting ready to submit to go to the R33 phase, fingers crossed. (Cohort 1, Project 6, Year 2)*
Assistance from NIDA staff	*I would say that working with [NIDA Project Otficer] has been great. [They’ve] been very responsive and very helpful with all the information in that transition. I personally struggle a lot with like legalese and all of the different government documents that are needed, and sometimes they have some conflicting information that I try to follow everything for, it’s precisely. And that kind of throws me through the loop. So maybe that’s just me. But there were some of those issues with the actual mechanism interactions and having somebody to go to get answers to questions like that was helpful. (Cohort 1, Project 4, Year 3)*
Assessment of Interactions with other projects	
Strengths	*They very intentionally have been trying to develop a community of researchers and projects. And part of that intentional community is a lot more interaction. Before, we would usually have with an R01 or some other research mechanism. In some ways, this has been great as far as sort of thinking about things from different perspectives. So, I do appreciate the intentional community. (Cohort 2, Project 2, Year 1)*
Limitations	*But I think some of that stuff is probably newer and more different for other grantees who don’t have D&I backgrounds, well and was challenging for us*
	*because we don’t have the direct clinical thing that we’re trying to implement or process that we’re trying to integrate into like ours is kind of a slight step back from that, our thing is the implementation strategy. So, it just meant that what we’re doing just didn’t quite map on. (Cohort 2, Project 3, Year 1)*
Data harmonization	*I haven’t seen anything substantive that addresses that harmonization across the projects. I mean, I have to say that the data reporting requirements now with NIH are rather onerous. And so, I think maybe there is some attempt to harmonize the data in that form, like when you report it to them in the final state. I don’t know about the other projects, but our project, it’s almost impossible for us to report the data, like the full data from our study, We will certainly be able to report our clinical trial data in some form. But how do we share an entire data [set]? Yeah. And I’ve become increasingly skeptical of the data aggregation efforts, I guess. (Cohort 2, Project 2, Year 2)*

**Table 4:** Reported satisfaction with assistance provided by the HD2A Support Centers

Year of grant funding	Helpful	TBD	Not needed/helpful
**RASC**			
Year 1 (n = 10/10)	5	4	1
Year 2 (n = 10/10)	6	3	2*
Year 3 (n = 6/6)	5	0	3*
**MERC**			
Year 1 (n = 10)	2	6	2
Year 2 (n = 10)	3	4	4*
Year 3 (n = 6)	2	3	1
**DISC**			
Year 1 (n = 10)	3	5	2
Year 2 (n = 10)	5	3	2
Year 3 (n = 6)	5	1	1*

* Projects reported support was helpful but required effort that was not helpful

## Data Availability

Data used in this study are available from the corresponding author upon reasonable request. All personal identifiers found in the data will be removed prior to sharing.
